# Call for ECDC Fellowship Programme cohort 2019

**DOI:** 10.2807/1560-7917.ES.2018.23.36.1809061

**Published:** 2018-09-06

**Authors:** Eurosurveillance editorial team

**Affiliations:** 1 European Centre for Disease Prevention and Control (ECDC), Stockholm, Sweden

The call for applications for the European Union-track cohort 2019 of the European Centre for Disease Prevention and Control (ECDC) Fellowship Programme is now open.

Interested candidates can apply to become a fellow in one of the two paths offered by the Programme: the European Programme for Intervention Epidemiology Training (EPIET) and the European Public Health Microbiology Training Programme (EUPHEM).

The deadline for application is **7 October 2018**.

The fellowship starts on **11 September 2019**.

For more information, please visit the ECDC website: https://ecdc.europa.eu/en/news-events/call-ecdc-fellowship-programme-epiet-euphem-cohort-2019.

